# Mapping peripheral and abdominal sarcopenia acquired in the acute phase of COVID-19 during 7 days of mechanical ventilation

**DOI:** 10.1038/s41598-023-29807-2

**Published:** 2023-03-02

**Authors:** Pedro Henrique de Moura, Helga de Souza, Daniella Cunha Brandão, Carlos Barros, Mario Correia, Cyda Reinaux, Wagner Souza Leite, Armele Dornelas de Andrade, Shirley Lima Campos

**Affiliations:** 1 Department of Physical Therapy, Federal University of Pernambuco (UFPE), 173, Aníbal Fernandes Avenue, Cidade Universitária, Recife, Pernambuco 50740-560 Brazil; 2 Hospital das Clínicas of the Federal University of Pernambuco (HC-UFPE), Recife, Pernambuco Brazil; 3 Hospital da Mulher do Recife (HMR), Recife, Pernambuc Brazil

**Keywords:** Diagnosis, Disease prevention, Medical imaging, Diseases

## Abstract

Our aim was to map acquired peripheral and abdominal sarcopenia in mechanically ventilated adults with COVID-19 through ultrasound measurements. On Days 1, 3, 5 and 7 after admission to critical care, the muscle thickness and cross-sectional area of the quadriceps, rectus femoris, vastus intermedius, tibialis anterior, medial and lateral gastrocnemius, deltoid, biceps brachii, rectus abdominis, internal and external oblique, and transversus abdominis were measured using bedside ultrasound. A total of 5460 ultrasound images were analyzed from 30 patients (age: 59.8 ± 15.6 years; 70% men). Muscle thickness loss was found in the bilateral anterior tibial and medial gastrocnemius muscles (range 11.5–14.6%) between Days 1 and 3; in the bilateral quadriceps, rectus femoris, lateral gastrocnemius, deltoid, and biceps brachii (range 16.3–39.1%) between Days 1 and 5; in the internal oblique abdominal (25.9%) between Days 1 and 5; and in the rectus and transversus abdominis (29%) between Days 1 and 7. The cross-sectional area was reduced in the bilateral tibialis anterior and left biceps brachii (range 24.6–25.6%) between Days 1 and 5 and in the bilateral rectus femoris and right biceps brachii (range 22.9–27.7%) between Days 1 and 7. These findings indicate that the peripheral and abdominal muscle loss is progressive during the first week of mechanical ventilation and is significantly higher in the lower limbs, left quadriceps and right rectus femoris muscles in critically ill patients with COVID-19.

## Introduction

Sarcopenia is a progressive and generalized disorder that affects the musculoskeletal system and is characterized by muscle mass, strength, and function losses related to inactivity, malnutrition, and the presence of critical illness^1, 2^. Studies show that prolonged bed rest is associated with multiple complications by inducing 25–33% muscle mass loss at an early stage of critical illness in the first week of hospitalization^3–6^. Furthermore, muscle impairment encompassing atrophy, weakness, or muscle function decrease has been widely reported (9–73%) in several studies and is persistent for approximately 6 months to 5 years after ICU discharge^7^.

Patients hospitalized with COVID-19 may require the use of mechanical ventilation, sedatives and/or neuromuscular blockers due to their illness severity, which would therefore require them to spend during their time in the ICU^8, 9^. Although common, the use of neuromuscular blockade is associated with a high risk of developing polyneuropathy in critically ill patients, thus negatively affecting their functional status^8, 10–12^. Critically ill patients affected by COVID-19 and under mechanical ventilation support may present with musculoskeletal impairments, with the main symptoms being myalgia, muscle fatigue, exercise intolerance, and myositis/rhabdomyolysis. symmetric neuropathy, critical illness myopathy and neuropathy^9, 13–15^.

Early detection of muscle changes is necessary for appropriate management of rehabilitation strategies^7, 16^, but volitional tests are not applicable in this acute disease phase^17, 18^. Nonvolitional assessment tools such as computed tomography and magnetic resonance imaging and invasive techniques such as biopsy, electromyography and nerve conduction studies are highly accurate for the diagnosis of muscle changes; however, these resources are limited in this specialized COVID-19 ICU scenario because of high cost, radiation exposure, and aerosol transmission-related health hazards^18–21^.

From this perspective, the assessment of muscle mass using ultrasound (USG) has been proven to be an effective method for identifying peripheral and abdominal muscle changes during critical illness, in addition to being a low-cost, nonionizing, noninvasive bedside assessment in uncooperative or unconscious patients, enabling screening for alterations in skeletal muscle structure^18, 22–26^. The literature is incipient on screening muscle damage, and there is no mapping of muscle mass loss to facilitate the tailoring of treatment^27, 28^. Recently, published studies regarding ultrasonography assessment in patients with COVID-19 were limited in that these studies only reported data on quadriceps and diaphragm measurements^29, 30^.

Thus, considering the impairment of the musculoskeletal system in these patients^15, 31^, a broader overall assessment approach is necessary to elucidate the progression of muscle mass loss to direct strategies to minimize or revert the affected muscles. Therefore, this study aims to map peripheral and abdominal sarcopenia acquired in the acute phase of COVID-19 through ultrasound measurements of muscle thickness and cross-sectional area of multiple muscles in critically ill patients on invasive mechanical ventilation during the first week of hospitalization in the ICU.

## Materials and methods

### Study design and configuration

This observational and longitudinal study was carried out at a single center, COVID-ICU Hospital da Mulher do Recife, between March and October 2020. It was approved by the institutional Research Ethics Committee of the Federal University of Pernambuco (No. 5.180.364). It was conducted in accordance with the 1964 Helsinki Declaration. The informed consent statement was obtained by phone from the legal representatives of the participants.

### Participants

Patients 18 years and older who were mechanically ventilated for more than 48 h and had a confirmed diagnosis of COVID-19 by RT‒PCR were included. Patients with neurological impairment, known myopathies, limb amputation or bedridden prior to admission were excluded.

### Sample calculation

The sample size calculation was performed using the G* Power version 3.1.9.4 statistical software program from the first ten medical data records included in the study. The means and standard deviations between D1 (first day) and D3 (third day) in the ICU were used, with α = 0.05 and β = 0.80 for muscle thickness and cross-sectional area of the muscle groups of interest (Online Resource S1).

### Procedures

The sample characterization data collected were age, sex, and presence of comorbidities (systemic arterial hypertension, diabetes mellitus, asthma, obesity, chronic obstructive pulmonary disease, heart disease, acute or chronic kidney disease, HIV infection), extubation, tracheostomy, ICU length of stay, and mortality. The Simplified Acute Physiology Score 3 (SAPS 3) and the Acute Physiology and Chronic Health Evaluation II (APACHE II) scale were also calculated within the first 24 h of the ICU stay.

All patients with COVID-19 were under sedation and mechanically ventilated in the supine position, according to the routine hospital practice. The patients presenting with refractory hypoxemia, hypercapnia, or patient-ventilator asynchrony were elected to additionally receive neuromuscular blockade (rocuronium bromide), which was infused at a dose of 9–12 µg/kg/min intravenously.

**Figure 1:**
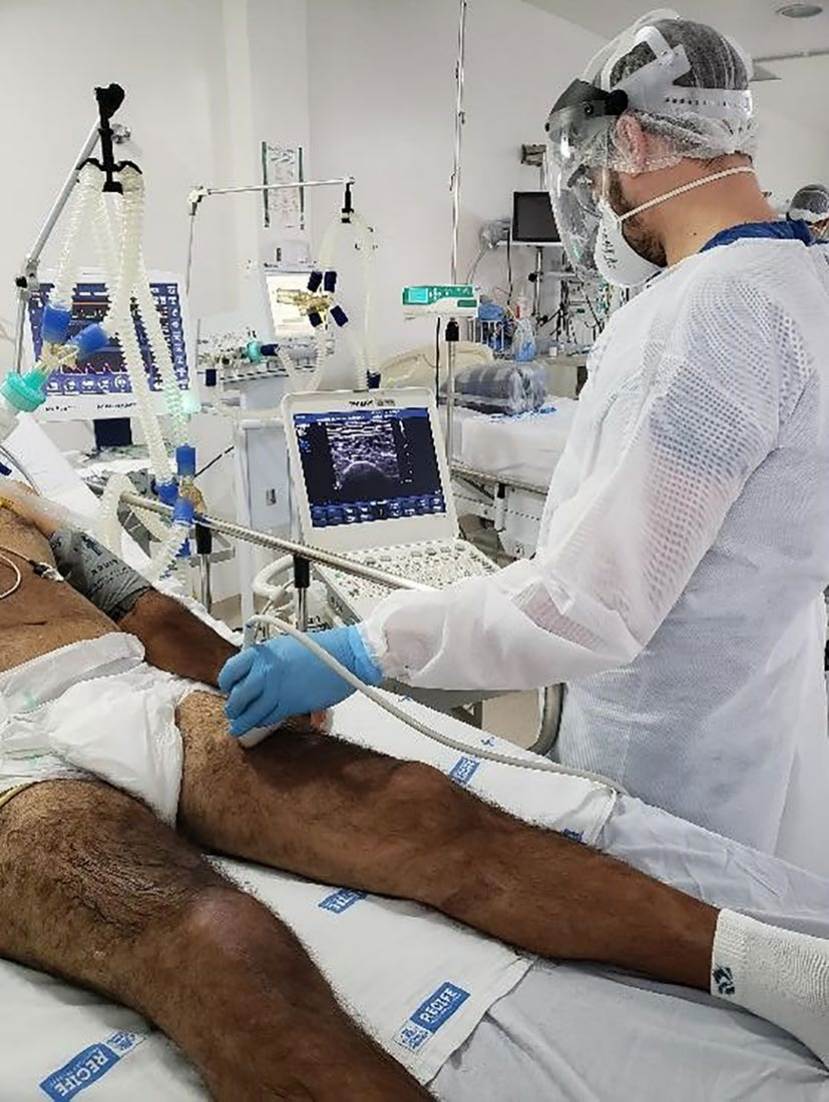
Ultrasound evaluation in the intensive care unit.

All ultrasonography of the peripheral and abdominal muscles was performed following the standard operating protocols (SOP) from the several references that are listed in Online Resource S2. The ultrasound scans were performed by a single assessor (Fig. 1) with a master’s degree in physical therapy and years of experience in ultrasound assessment in critically ill patients. He underwent a couple years of training during his PT Emergency and Trauma residency program. His training consisted of technical lectures (HS; SLC) and individual training sessions from a formal ultrasound physiotherapist/researcher specialized in clinical musculoskeletal ultrasound (HS). Throughout the training period, a few US assessment sessions were supervised by HS. A pilot study revealed that the intra-assessor ICC and limits of agreements were strong and reliable. The same methodology was applied in the study of Willemse et al.^32^.

To ensure tracking and reliability of the measurements, the assessor performed the US measurements immediately on the ICU admission day by using a Philips ultrasound machine (CX50, Washington. United States), with an 8.0 MHz linear probe (L12-3 transducer, Washington. United States). The patients’ ultrasound measurements were also registered on their medical records for further reassessments.

Muscle mass was determined by determining muscle thickness (mm) and cross-sectional area (cm^2^) in the brightness mode (B-mode) of the US. The scanning depth was set to 3.5 cm, and the selected region was set to include as much muscle as possible, excluding surrounding bone or fascia. Time gain and compensation were set to the neutral position. The focus number and area were maximally increased to remain consistent across all participants and to adjust for differences in muscle size between them. The following variables were analyzed:

- Muscle thickness of the lower limbs (bilaterally): quadriceps, rectus femoris, vastus intermedius, tibialis anterior, medial and lateral gastrocnemius muscles, for which imaging was achieved by flexion at the knee with the foot placed flat onto the patient’s bed.
- Muscle thickness of the upper limbs (bilaterally): deltoid and biceps brachii muscles;
- Abdominal muscle thickness: rectus abdominis, internal and external oblique, and transversus abdominis muscles;
- Cross-sectional area (bilaterally): rectus femoris, biceps brachii, and tibialis anterior muscles

The measurements of Day 1, denoted by the first 24–48 h of mechanical ventilation, were considered the baseline to be compared with the follow-up measurements of Days 3, 5 and 7 from ICU admission.

### Statistical analysis

Descriptive and inferential analyses were conducted using the Statistical Package for the Social Sciences (SPSS) program for Windows (version 26.0. Chicago. IL), and a significance level of p < 0.05 was established.

The percentage change (Change%) for the values obtained for muscle thickness and the cross-sectional area between Days 1–3, 1–5, and 1–7 of MV were also calculated using the expression (Change%) = [(Final value − Initial value)/initial value] × 100 and the coefficient of variation (CV) given by the ratio between standard deviation/mean × 100 for all muscle groups. Thickness and cross-sectional area measurements for each muscle group between Days 1, 3, 5 and 7 were compared by analysis of variance of repeated measures (Online Resources S3, S4, S5, S6 and S7).

#### Ancillary analysis

1. Considering the use of neuromuscular blockade as a presumed bias or confounding variable, a sensitivity analysis was performed comparing the sample divided into two groups by the use of neuromuscular blockade, 15 in each group. The baseline characteristics between the groups were compared using the t test for independent samples for variables with a normal distribution and the Mann‒Whitney test for variables with a nonnormal distribution. Chi-square Test was used to compare proportions and corrected by Fisher's exact test (if the number of cases < 5). An additional analysis was performed to verify the effect of NMB on muscle thickness and cross-sectional area alterations in a multivariate analysis of variance (MANOVA) test with NMB as a covariate in the model (Online Resources S8 and S9).
2. Binomial tests were used to compare the proportions of muscle thickness loss, marked by a cutoff higher than 15% (yes/no), and of a cross-sectional area decline marked by a cutoff higher than 12% (yes/no) between the observed and expected observations set with 0.50 in each category (Online Resource S10).
3. To verify the association between muscle thickness loss, marked by a cutoff higher than 15% (yes/no) and of a cross-sectional area decline marked by a cutoff higher than 12% (yes/no), and the categorical outcomes (ICU discharge/death), we used the chi-squared test corrected by Fisher's exact test and assessed the risk by determining the odds ratio. (Online Resource S11)
4. Three automatic linear models for ICU discharge/death were run to analyze the influence of (1) muscle mass thickness loss (higher than 15%) and cross-sectional area decline (higher than 12%); (2) percentage change of muscle mass and cross-sectional area, and (3) risk factors. These automatic models were generated by employing the following settings: the selection method was “Forward Stepwise”, and the criteria for entry/removal: Information Criterion (AICC) including effects with p values less than 0.05 and the removal of effects with p values greater than 0.1. The model selected was the one with the highest adjusted R^2^ and the most significant change in the F value. The settings to determine the behavior of the ensembled model that was employed were the “mean” as a default combining the rules for continuous targets on large datasets and for boosting and bagging, the number of base models to build was set as 10. (Online Resource S12)

### Ethics approval and consent to participate

This study followed the National Health Council (Resolution 466/2012) and the Declaration of Helsinki for research on human beings and was approved by the research ethics committee of the institution. The hospital also consented to the research.

## Results

The sample consisted of 30 patients with 5,460 images analyzed. This total number of images corresponded to the following details: three measurements were performed on each body segment (total of 60 measurements), 12 patients were assessed twice (total of 480), 5 patients were assessed 3 times (total of 300) and 13 patients were assessed four times (total of 1040). The lowest ICC was 0.97 with a confidence interval of 0.91–0.99 in biceps brachii thickness.

The baseline characteristics of the patients included are shown in Table 1. A total of 70% were males aged between 25 and 86 years (56.7% were > 60 years) and were seriously ill according to APACHE II and SAPS 3. The patients generally had more than one comorbidity, with the most frequent being systemic arterial hypertension and diabetes mellitus (46.7% and 43.3%, respectively). The mortality in the ICU in the first week was 43.3% (13/30). Half of the patients received neuromuscular blockade, and they did not present with significant differences in their clinical characteristics, including illness severity, compared to those who received neuromuscular blockade (Online Resource S8).

**Table 1:** Baseline clinical characteristics of the patients.

Variables	Total (n = 30)
Male/female (N, %)	21/9 (70/30)
Age, years (mean ± SD)	59.8 ± 15.6
APACHE II (mean ± SD)	24.0 ± 6.1
SAPS 3 (mean ± SD)	64.17 ± 10.5
Mechanical ventilation use, days (mean ± SD)	5.0 ± 1.9
Neuromuscular blockade use (N, %)	15 (50)
Outcomes	
Extubation < 7 days (N, %)	5 (16.7)
Tracheostomy < 7 days (N, %)	3 (10)
Mortality < 7 days (N, %)	13 (43.3)
Comorbidities	
Systemic hypertension (N, %)	14 (46.7)
Diabetes mellites (N, %)	13 (43.3)
Asthma (N, %)	2 (6.7)
Obesity (N, %)	8 (26.7)
COPD (N, %)	3 (10)
Heart diseases (N, %)	4 (13.3)
Kidney diseases (N, %)	3 (10)
Other respiratory diseases (N, %)	2 (6.7)
Human immunodeficiency virus (N, %)	1 (3.3)

*APACHE II* acute physiology and chronic health evaluation II, *SAPS 3* simplified acute physiology score 3, *COPD* chronic obstructive pulmonary disease, *HIV* human immunodeficiency virus.

**Figure 2:**
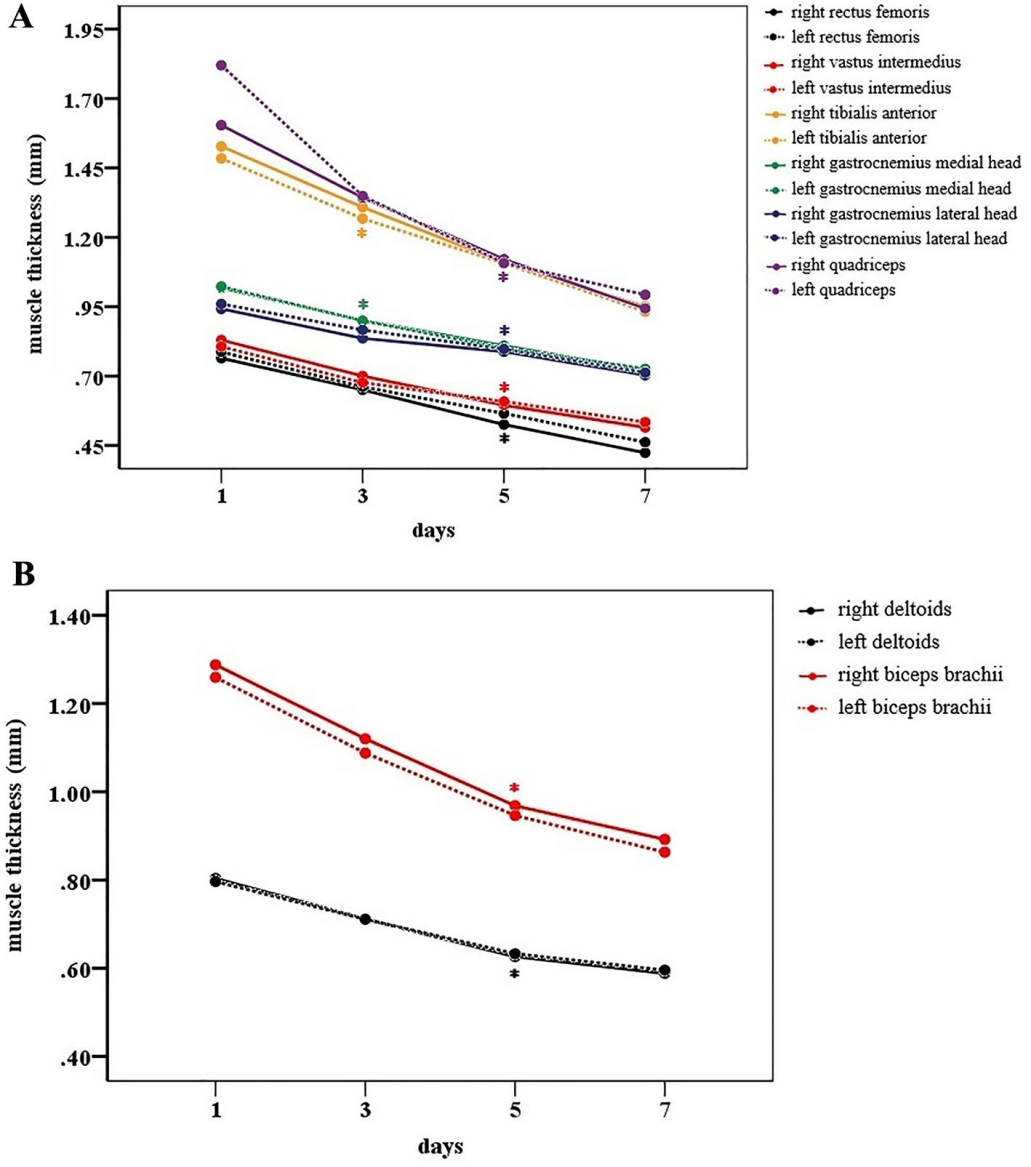
Thickness of peripheral muscles over 7 days. (**A**) Thickness of lower limb muscles. Significant differences between the corresponding day and Day 1 are indicated by the signal *: between D1-D3 for the right (p = 0.017) and left medial gastrocnemius muscle (p = 0.018); right (p = 0.018) and left tibialis anterior muscle (p = 0.006); between D1-D5 for the right (p = 0.003) and left quadriceps muscle (p = 0.017); the right (p = 0.016) and left rectus femoris muscle (0.033); the right (p = 0.012) and left vastus intermedius muscle (p = 0.05); and the right (p = 0.015) and left lateral gastrocnemius muscle (p = 0.003). In (**B**) the thickness of upper limb muscles. Significant differences between the corresponding day and Day 1 are indicated by the signal *: between D1-D5 for the right (p = 0.011) and left deltoid muscle (p = 0.014) and the right (p = 0.007) and left biceps brachii (p = 0.001).

The muscle thickness and cross-sectional area data for all muscle groups and follow-up analyses are presented in the Supplementary material S3 to S7. There was a progressive loss of muscle thickness of the lower limbs over time, with a significant decline from the 3rd day of the ICU stay for the right (D1 = 1.018 vs. D3 = 0.901; p = 0.017) and left medial gastrocnemius muscles (D1 = 1.023 vs. D3 = 0.899; p = 0.018) and the right (D1 = 1.528 vs. D3 = 1.308; p = 0.018) and left anterior tibial muscles (D1 = 1.484 vs. D3 = 1.267; p = 0.006) (Fig. 2A).

The other muscles of the lower limbs were affected from the 5th day in the ICU, with a more significant loss of muscle mass in the right (D1 = 1.604 vs. D5 = 1.121; p = 0.003) and left quadriceps muscles (D1 = 1.820 vs. D5 = 1.107; p = 0.017). On the fifth day, the impacts on the muscles were as follows: right (D1 = 0.764 vs. D5 = 0.526; p = 0.016) and left rectus femoris (D1 = 0.787 vs. D5 = 0.565; 0.033); right (D1 = 0.830 vs. D5 = 0.595; p = 0.012) and left vastus intermedius (D1 = 0.806 vs. D5 = 0.608; p = 0.05); and right (D1 = 0.942 vs. D5 = 0.708; p = 0.015) and left lateral gastrocnemius (D1 = 0.955 vs. D5 = 0.794; p = 0.003) (Fig. 2A).

The deltoid and biceps muscles bilaterally showed a progressive loss of thickness over time, with a significant decline from the 5th day of evaluation: the right (D1 = 0.803 vs. D5 = 0.626; p = 0.011) and left deltoid (D1 = 0.797 vs. D5 = 0.633; p = 0.014) and the right (D1 = 1.288 vs. D5 = 0.968; P = 0.007) and left biceps (D1 = 1.259 vs. D5 = 0.946; p = 0.001) (Fig. 2B).

**Figure 3:**
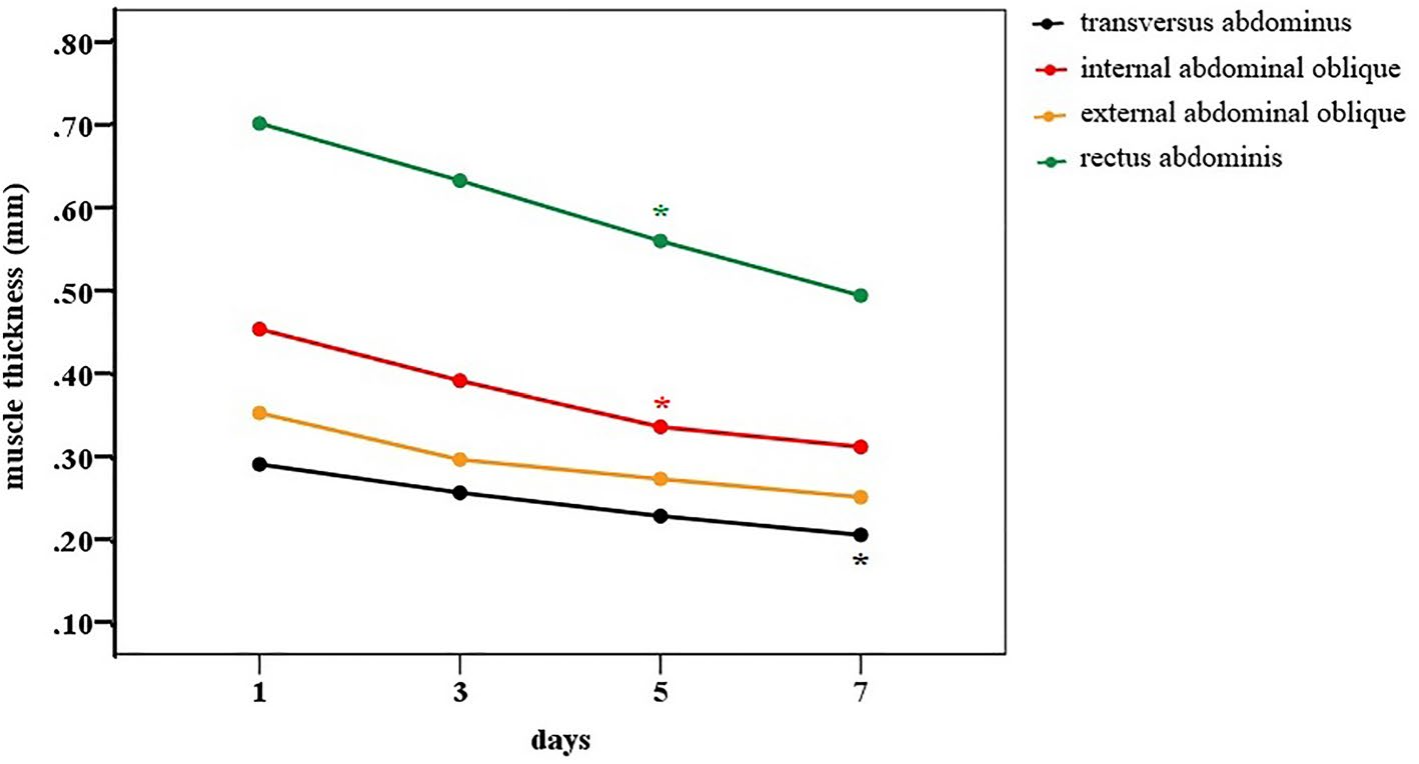
Thickness of abdominal muscle mass over 7 days from ICU admission. Significant differences between the corresponding day and Day 1 are indicated by the signal *: between D1-D5 for the rectus abdominis muscle (p = 0.037) and internal oblique muscle (p = 0.004) and between D1-D7 for the transversus abdominis muscle (p = 0.024).

The abdominal muscles showed progressive loss of thickness between the fifth and seventh day of MV in the ICU. The rectus abdominis muscle had a significant decline from the 5th day of evaluation (D1 = 0.701 vs. D5 = 0.560; p = 0.037), as well as the internal oblique (D1 = 0.453 vs. D5 = 0.336; p = 0.004). The transversus abdominis muscle (D1 = 0.290 vs. D7 = 0.205; p = 0.024) only showed a decline in muscle thickness from the 7th day of evaluation, while the external oblique muscle showed a reduction in mass at the end of the 7 days of MV, but without a significant difference (D1 = 0.353 vs. D7 = 0.251; p = 0.096) (Fig. 3).

Once the process of muscle loss in the lower, upper and abdominal limbs began on the 3rd or 5th day, it progressed until the end of the first week (Figs. 2 and 3). Online Resources S3–S7).

**Figure 4:**
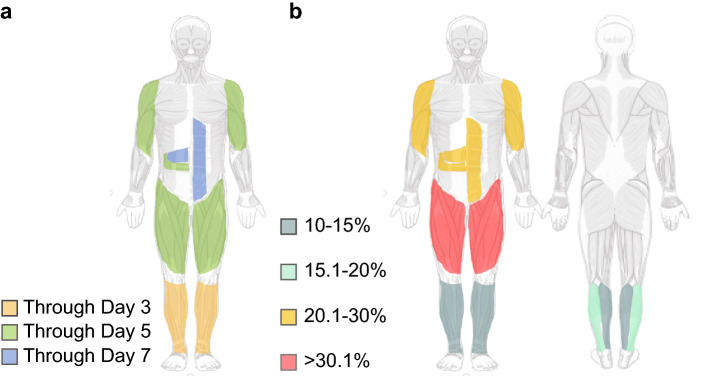
Mapping peripheral and abdominal sarcopenia acquired in the acute phase of COVID-19 during 7 days of mechanical ventilation. In (**a**), the pattern of muscle loss measured by ultrasound over 7 days is shown. It is observed that there is a loss of up to 3 days in the lower limbs distally (tibial anterior and medial gastrocnemius) that evolves over 5 days from the lower limbs (quadriceps. rectus femoris. vastus intermedius and lateral gastrocnemius) to the upper limbs (biceps and deltoid) and rectus abdominis. Finally, after 7 days, the abdominal muscles (transversus abdominis and internal oblique) are affected. In (**b**), the percentage of loss of muscle thickness can be quantitatively observed. with the quadriceps and rectus femoris being more affected (> 30.1%); deltoid. biceps brachii. internal oblique. rectus abdominis and transversus abdominis (20.1% to 30%); lateral gastrocnemius (15.1% to 20%); tibialis anterior and medial gastrocnemius (10% to 15%). Source: smart.servier.com.

The described results reveal a reduction pattern in muscle thickness, initially affecting the lower limbs and then the upper and abdominal limbs with the body map shown in Fig. 4a. The left quadriceps and right rectus femoris muscles showed the greatest variations in muscle thickness percentage loss at the end of 7 days (45.44% and 44.54%., respectively). The smallest variation was observed for the left deltoid muscle (25.28%). The thickness loss percentage change (Change%) map for all muscles is shown in Fig. 4b.

**Figure 5:**
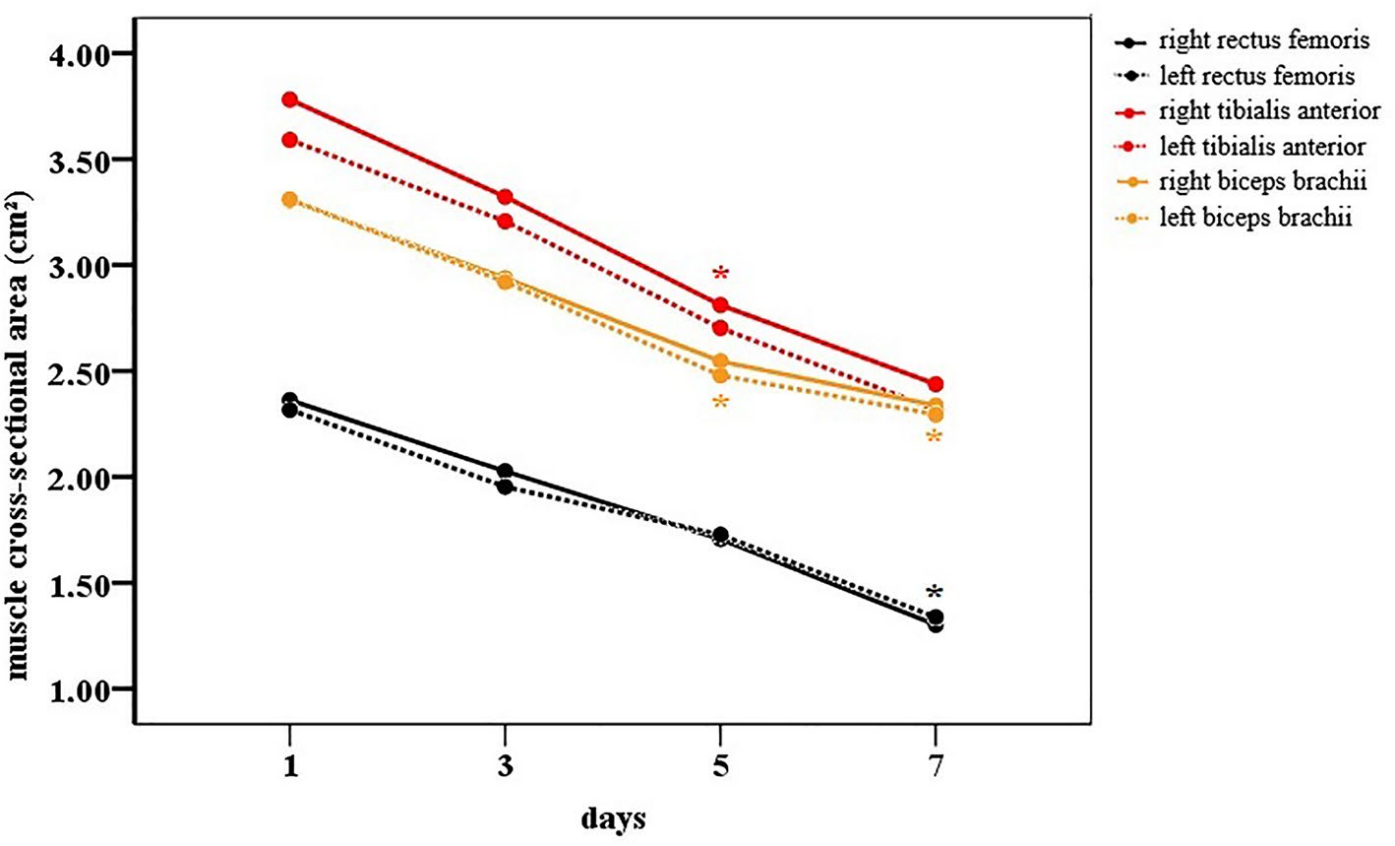
Cross-sectional area over 7 days in the ICU. Significant differences between the corresponding day and Day 1 are indicated by the signal *: between D1-D5 for the right (p = 0.006) and left tibialis anterior muscle (p = 0.003); left biceps brachii (p = 0.04); between D1-D7 for the right (p = 0.004) and left rectus femoris muscle (p = 0.005); right biceps brachii (p = 0.035).

The evaluation results after considering the cross-sectional area showed that the reduction of this measure occurred from the 5th day onward for the right (D1 = 3.781 vs. D5 = 2.811; p = 0.006) and left anterior tibialis muscles (D1 = 3.569 vs. D5 = 2.688; p = 0.003) and left biceps brachii (D1 = 3.309 vs. D5 = 2.479; p = 0.04). This loss pattern for the other muscles that were evaluated was observed only from the 7th day of ICU stay (Fig. 5), with the right (D1 = 2.362 vs. D7 = 1.301; p = 0.004) and left rectus femoris (D1 = 2.317 vs. D7 = 1.337; p = 0.005) and right biceps brachii (D1 = 3.305 vs. D7 = 2.337; p = 0.035). The percentage change (Change%) in the cross-sectional area loss is shown in the Supplementary material (Online Resource  S7).

MANOVA showed that there was an effect of neuromuscular blockade on the decline in muscle thickness [Pillai trace = 0.576; F (20. 67) = 4.54; p < 0.001] and on the reduction of the cross-sectional area [Pillai trace = 0.255; F (6.81) = 4.62; p < 0.001]. Details are presented in the supplementary material (Online Resource S9).

## Discussion

The main findings of this study were as follows:

1. There was muscular architecture remodeling marked by a decrease in the muscle thickness and cross-sectional area of the peripheral and abdominal muscles, aggravated by prolonged immobility associated with mechanical ventilation, in the acute phase of COVID-19 critical illness.
2. The muscle mass loss followed a caudal-cranial direction over the 7 days of ICU stay while the patient was under mechanical ventilation. The lower limbs showed greater loss within the first 3 days and progressively declined by Day 7. Meanwhile, the upper limbs showed a substantial decline in mass after Day 5, and the abdominal muscle mass declined by Day 7.
3. The muscle thickness and cross-sectional area measurements enabled screening for muscle wasting and ICU-acquired sarcopenia in the acute phase of COVID-19.

Muscle disuse is an important risk factor for critically ill patients with COVID-19 who require admission to the ICU and mechanical ventilation, as observed in the study by Andrade-Junior^29^. They observed a reduction by 30.1% in the CSA of the rectus femoris and by 18.6% in the anterior quadriceps thickness 10 days after ICU admission. However, our study has shown even greater muscle mass loss within the first 7 days, indicated by the reduction in CSA of the right (44.93%) and left (42.28%) rectus femoris, as well as in the thickness of the right (41.18%) and the left (45.44%) quadriceps.

Skeletal muscle disuse caused by prolonged bed rest leads to muscle fiber atrophy. Fast-twitch fibers (type II) are more sensitive to the inflammatory process from critical illness and are the most affected^33^. They may undergo a process of necrosis and be replaced by adipose tissue or fibrosis, directly compromising the activities of daily living performance, especially the power activities in which these fibers are most employed.^33–35^.

Previous studies have reported that muscle tissue injury may be associated with damage regulated by proinflammatory cytokines in patients with COVID-19^15, 30^. Furthermore, some authors claim that the COVID-19 virus binds to the angiotensin-converting enzyme 2 (ACE 2) receptor in human cells, and this virus has high expression in the musculoskeletal system, thus causing direct invasion by hematogenous dissemination^15, 36^.

Evidence of body composition assessment in non-COVID-19 patients has demonstrated protein loss because of proteolysis. This alteration can be explained by the imbalance of protein metabolism since the skeletal muscle system is considered a protein reservoir. Protein synthesis in the critically ill is severely low and is equivalent to the levels found during fasting on the first day of illness, followed by an increase to normal levels by the end of the first week. However, a catabolic state that manifests clinically as weight loss, undernutrition and sarcopenia remains during the ICU stay despite nutritional support^37, 38^.

To date, no study has tracked sarcopenia ultrasonographically in critically ill patients with COVID-19 in the acute phase. General studies have reported that muscle mass loss in the lower limbs of critically ill patients is prevalent^39^. Our findings showed that this mass declination followed a caudal-cranial pattern that was detected on the 3rd day in the lower limbs and then was detected in the upper limbs on the 5th day, maintaining a progressive loss. The disparity in sarcopenia in the skeletal muscle of the upper and lower limbs has not been fully elucidated but is speculated to be the result of less physical activity^40–42^, especially lower-limb disuse, as the upper limbs are constantly utilized for activities of daily living across the patient’s lifespan^43^.

Another possible explanation is that SARS-CoV-2 infection affects neural tissue, therefore inducing neural cell apoptosis and consequently affecting muscle function^44^. Some authors report similarities between this infection pathophysiology and that of demyelinating diseases, such as Guillain‒Barré Syndrome and acute myelitis, which present as a pattern of muscle weakness starting from the lower to upper extremities^45, 46^. We could not assess nerve conduction in our sample, thus limiting the ability to obtain stronger results.

A recent Italian study analyzing the quality of peripheral and respiratory muscle ultrasound characteristics in mechanically ventilated patients at Days 3 and 7 found a significant change in the rectus femoris and quadriceps by Day 7^47^. In our study, this rectus femoris mass declination was observed even sooner (Day 5) and was observed bilaterally partially due to methodological reasons.

Neuromuscular blockade was used to facilitate oxygenation or mechanical ventilation coupling in patients with COVID-19. It is known that the use of this resource was associated with a 25% greater chance of developing ICU-acquired muscle weakness due to the deregulation of acetylcholine metabolism and upregulation of receptor subtypes that are less sensitive to acetylcholine^48, 49^. In our study, it was observed that there was a statistically significant effect of the use of neuromuscular blockers in reducing muscle thickness and cross-sectional area.

Other factors need to be considered in muscle thickness reduction, such as illness severity, age, sepsis and multiple organ failure^50^. This reduction can also be masked by fluid overload represented by positive fluid balance, as well as by metabolic alterations associated with inadequate nutritional support^51^ and by imbalances in calcium ion homeostasis (Ca ^+ +^)^37, 51, 52^.

Ultrasound screening for muscle mass loss is a valuable asset to anticipate muscle dysfunction in critically ill patients with COVID-19 under mechanical ventilation and sedation. The diagnosis of ICU-acquired weakness and functional dysfunctions are usually detected late after individual awakening and cooperation with functionality assessments. By identifying individuals who may develop future deterioration of strength and functionality early, strategies can be set to prevent or attenuate the muscle damage induced by extended bed rest and the use of mechanical ventilation in the acute phase of COVID-19 critical illness.

Some limitations that are present in this study need to be addressed. First, the single-centered feature can limit the generalization of our results. Second, unadjusted confounding variables such as age, gender, fluid balance and albumin may result in the over- or underestimation of the ultrasound-related outcomes, although we believe that they would not change the overall results obtained, which showed to be robust. Third, it was not possible to retrieve baseline assessments of the onset of COVID-19-related symptoms before the patients were admitted to the ICU, as this timing can have some influence on the outcomes.

This is a pioneering study in tracking peripheral and abdominal muscle wasting via ultrasound in critically ill patients with COVID-19 and is thereby clinically relevant for guiding therapeutic interventions to promote functional recovery as early as possible through an individualized approach.

## Conclusion

Peripheral and abdominal muscle mass loss is progressive over the ICU time of mechanically ventilated patients with COVID-19. The lower limbs, left quadriceps and right rectus femoris muscles showed greater muscle wasting during the first week.

## Supplementary Information

Supplementary Information.

## Data Availability

The datasets used and/or analyzed during the current study are available from the corresponding author on reasonable request.
