# Supplementary material for: Mapping peripheral and abdominal sarcopenia acquired in the acute phase of COVID-19 during 7 days of mechanical ventilation
Source: Sci Rep. 2023 Mar 2;13:3514. doi: 10.1038/s41598-023-29807-2 (PMC9978280; doi:10.1038/s41598-023-29807-2)
Supplement: Supplementary file 1 — Supplementary Information. [file 41598_2023_29807_MOESM1_ESM.docx]

**Supplementary Material**

**Mapping Peripheral and Abdominal Sarcopenia Acquired in the Acute Phase of Covid-19 During 7 Days in Mechanical Ventilation.**

Pedro Henrique de Moura^1^; Helga de Souza^2^; Daniella Cunha Brandão^1^ Carlos Barros^3^; Mario Correia^3^; Cyda Reinaux^1^; Wagner Souza Leite^1^, Armele Dornelas de Andrade ^1^; Shirley Lima Campos^1*^

^1^ Department of Physical Therapy. Federal University of Pernambuco (UFPE). 173, Aníbal Fernandes Avenue, Cidade Universitária, Recife, Pernambuco. 50740-560. Brazil.

^2^ Hospital das Clínicas of the Federal University of Pernambuco (HC-UFPE). Recife. Pernambuco. Brazil.

^3^ Hospital da Mulher do Recife (HMR). Recife, Pernambuco, Brazil.

***Corresponding author:** Shirley Lima Campos. Federal University of Pernambuco. Department of Physiotherapy. 173. Aníbal Fernandes Avenue, Cidade Universitária, 50740-560. Recife. Pernambuco. Brazil. Email address: [shirley.campos@ufpe.br](mailto:shirley.campos@ufpe.br). https://orcid.org/0000-0003-3079-8300.

**Online Resource S1**

**Sample calculation with effect size for the analyzed variables**

The sample size calculation was performed for the 26 muscle groups. The largest effect size was 1.83 for the variable muscle thickness of the right biceps brachii, estimating a sample of 5 subjects and the smallest effect size was for the variable muscle thickness of the transversus abdominis, with a sample size of 1.204 subjects.

Thus, an estimated sample of 30 subjects was adopted for this study, capable of reaching a high enough number for most of the variables of this study, with the exception of the muscle thickness of the left lateral gastrocnemius, right deltoid, external oblique, transversus abdominis and the cross-sectional area of the left tibialis anterior variables

**Table S1. Sample calculation.**

| **Outcome**  **Muscle thickness (cm)** | **D1**  **Mean ± SD** | **D3**  **Mean ± SD** | **n** | **Correlation** | **Effect size** | **Sample range** |
| --- | --- | --- | --- | --- | --- | --- |
| **Right quadriceps** | 1.53 ± 0.68 | 1.35 ± 0.64 | 14 | 0.95 | 0.83 | yes |
| **Left quadriceps** | 1.54 ± 0.62 | 1.31 ± 0.60 | 16 | 0.88 | 0.77 | yes |
| **Right rectus femoris** | 0.75 ± 0.38 | 0.68 ± 0.35 | 29 | 0.94 | 0.54 | yes |
| **Left rectus femoris** | 0.74 ± 0.29 | 0.62 ± 0.24 | 20 | 0.79 | 0.67 | yes |
| **Right vastus intermedius** | 0.76 ± 0.33 | 0.68 ± 0.30 | 21 | 0.93 | 0.66 | yes |
| **Left vastus intermedius** | 0.78 ± 0.36 | 0.64 ± 0.34 | 11 | 0.92 | 0.99 | yes |
| **Right tibialis anterior** | 1.53 ± 0.36 | 1.39 ± 0.39 | 29 | 0.77 | 0.55 | yes |
| **Left tibialis anterior** | 1.37 ± 0.24 | 1.24 ± 0.26 | 25 | 0.61 | 0.59 | yes |
| **Right medial gastrocnemius** | 1.00 ± 0.18 | 0.91 ± 0.17 | 18 | 0.74 | 0.71 | yes |
| **Left medial gastrocnemius** | 0.98 ± 0.22 | 0.88 ± 0.20 | 13 | 0.86 | 0.89 | yes |
| **Right lateral gastrocnemius** | 0.92 ± 0.20 | 0.83 ± 0.17 | 13 | 0.86 | 0.88 | yes |
| **Left lateral gastrocnemius** | 0.89 ± 0.14 | 0.86 ± 0.17 | 68 | 0.86 | 0.34 | no |
| **Right deltoid** | 0.81 ± 0.26 | 0.76 ± 0.22 | 33 | 0.93 | 0.51 | no |
| **Left deltoid** | 0.76 ± 0.26 | 0.70 ± 0.22 | 9 | 0.99 | 1.14 | yes |
| **Right biceps brachii** | 1.23 ± 0.36 | 1.12 ± 0.32 | 8 | 0.97 | 1.19 | yes |
| **Left biceps brachii** | 1.21 ± 0.35 | 1.05 ± 0.32 | 5 | 0.97 | 1.83 | yes |
| **Rectus abdominis** | 0.81 ± 0.23 | 0.74 ± 0.17 | 16 | 0.94 | 0.77 | yes |
| **Internal oblique** | 0.51 ± 0.13 | 0.41 ± 0.14 | 12 | 0.67 | 0.91 | yes |
| **External oblique** | 0.35 ± 0.15 | 0.32 ± 0.13 | 74 | 0.80 | 0.33 | no |
| **Transversus abdominis** | 0.30 ± 0.68 | 0.27 ± 0.53 | 1204 | 0.84 | 0.08 | No |
| **Outcome**  **Cross-sectional area (cm^2^)** | **D1**  **Mean ± SD** | **D3**  **Mean ± SD** | **n** | **Correlation** | **Effect size** | **Sample range** |
| **Right rectus femoris** | 2.40 ± 1.64 | 2.16 ± 1.61 | 10 | 0.99 | 1.03 | yes |
| **Left rectus femoris** | 2.25 ± 1.42 | 1.95 ± 1.43 | 13 | 0.97 | 0.86 | yes |
| **Right tibialis anterior** | 3.99 ± 1.56 | 3.57 ± 1.40 | 8 | 0.98 | 1.25 | yes |
| **Left tibialis anterior** | 3.57 ± 1.08 | 3.38 ± 0.98 | 64 | 0.87 | 0.36 | no |
| **Right biceps brachii** | 3.39 ± 1.27 | 3.01 ± 1.25 | 8 | 0.97 | 1.23 | yes |
| **Left biceps brachii** | 3.49 ± 1.28 | 2.96 ± 1.23 | 7 | 0.95 | 1.35 | yes |

**Notes:** cm: centimeters; cm^2^: square centimeters.

**Online Resource S2**

**Table S2. Standard operating protocol (SOP) adopted at the institution to assess peripheral and abdominal muscles with ultrasound.**

| **Muscle group** | **Ultrasonography transducer location**  **Description** |
| --- | --- |
| Quadriceps (rectus femoris and vastus intermedius)  *Witteveen et al. 2017* | 1/2 between the anterior superior iliac spine and base of the patella with the lower limb in knee extension (neutral position) and supported on the stretcher. |
| Tibialis anterior  *Turton et al. 2016* | 1/3 between the edge of the patella and lateral malleolus with the lower limb in knee extension (neutral position) and supported on the stretcher. |
| Gastrocnemius (medial and lateral)  *Turton et al. 2016* | 1/3 between the popliteal surface and insertion of the Achilles tendon with the lower limb in triple flexion and foot resting on the bed. |
| Deltoid  *Yang et al. 2018* | 1/2 between the acromion and deltoid tuberosity with the upper limb in anatomical position and supported on the stretcher |
| Biceps brachii  *Witteveen et al. 2017* | 2/3 between the acromion and antecubital sulcus with the upper limb in anatomical position and supported on the stretcher. |
| Rectus abdominis  *Shi et al. 2019* | 2-3 centimeters above the umbilicus with the patient lying supine with head elevated at 30°. |
| Oblique (external and internal) and transverse abdominis  *Shi et al. 2019* | Perpendicular line between the anterior superior iliac spine and umbilical line with the patient lying supine and head elevated at 30°. |

1. Turton, P., Hay, R., Taylor, J., McPhee, J. & Welters, I. Human limb skeletal muscle wasting and architectural remodeling during five to ten days intubation and ventilation in critical care - an observational study using ultrasound. *BMC Anesthesiol* **16**, 119 (2016).

2. Witteveen, E. *et al.* Diagnostic accuracy of quantitative neuromuscular ultrasound for the diagnosis of intensive care unit-acquired weakness: a cross-sectional observational study. *Ann Intensive Care* **7**, 40 (2017).

3. Yang, C. *et al.* Musculoskeletal Ultrasonography Assessment of Functional Magnetic Stimulation on the Effect of Glenohumeral Subluxation in Acute Poststroke Hemiplegic Patients. *Biomed Res Int* **2018**, 6085961 (2018).

4. Shi, Z.-H. *et al.* Expiratory muscle dysfunction in critically ill patients: towards improved understanding. *Intensive Care Med* **45**, 1061–1071 (2019).

**Online Resource S3**

**Table S3. Evolution of muscle thickness in lower limb muscles of mechanically ventilated patients with Covid-19 in the first week of admission to ICU.**

|  | **Right lower limb** | | | | **Left lower limb** | | | |
| --- | --- | --- | --- | --- | --- | --- | --- | --- |
| **Muscle thickness (cm)** | **Mean** | **95% CI** | **CV (%)** | ***p*** | **Mean** | **95% CI** | **CV (%)** | ***p*** |
| Quadriceps |  |  |  |  |  |  |  |  |
| D1 | 1.604 | (1.442-1.766) | 33.15 |  | 1.820 | (1.533-2.107) | 67.77 |  |
| D3 | 1.343 | (1.181-1.505) | 36.00 | 0.115 | 1.349 | (1.181-1.505) | 35.74 | 0.105 |
| D5 | 1.121 | (0.912-1.330) | 28.27 | **0.003** | 1.107 | (0.912-1.330) | 35.18 | **0.017** |
| D7 | 0.943 | (0.697-1.190) | 24.94 | **<0.001** | 0.993 | (0.697-1.190) | 30.85 | **0.012** |
| Femoral rectus |  |  |  |  |  |  |  |  |
| D1 | 0.764 | (0.669-0.860) | 39.68 |  | 0.787 | (0.690-0.884) | 41.35 |  |
| D3 | 0.650 | (0.555-0.746) | 43.88 | 0.343 | 0.661 | (0.564-0.758) | 38.20 | 0.269 |
| D5 | 0.526 | (0.402-0.649) | 37.36 | **0.016** | 0.565 | (0.440-0.691) | 37.11 | **0.033** |
| D7 | 0.424 | (0.278-0.569) | 41.84 | **0.001** | 0.462 | (0.314-0.609) | 46.88 | **0.002** |
| Vastus intermedius |  |  |  |  |  |  |  |  |
| D1 | 0.830 | (0.739-0.921) | 35.09 |  | 0.806 | (0.714-0.898) | 35.99 |  |
| D3 | 0.700 | (0.610-0.791) | 35.87 | 0.192 | 0.676 | (0.584-0.768) | 38.16 | 0.203 |
| D5 | 0.595 | (0.478-0.712) | 35.25 | **0.012** | 0.608 | (0.489-0.727) | 34.92 | **0.050** |
| D7 | 0.515 | (0.377-0.653) | 35.36 | **0.002** | 0.534 | (0.394-0.674) | 36.76 | **0.009** |
| Medial gastrocnemius |  |  |  |  |  |  |  |  |
| D1 | 1.018 | (0.963-1.072) | 16.95 |  | 1.023 | (0.965-1.080) | 18.48 |  |
| D3 | 0.901 | (0.846-0.955) | 15.95 | **0.017** | 0.899 | (0.842-0.957) | 17.73 | **0.018** |
| D5 | 0.809 | (0.739-0.880) | 17.30 | **<0.001** | 0.801 | (0.726-0.875) | 15.81 | **<0.001** |
| D7 | 0.721 | (0.638-804) | 16.72 | **<0.001** | 0.726 | (0.639-0.814) | 15.46 | **<0.001** |
| Lateral gastrocnemius |  |  |  |  |  |  |  |  |
| D1 | 0.942 | (0.881-1.003) | 21.23 |  | 0.955 | (0.901-1.009) | 18.24 |  |
| D3 | 0.836 | (0.775-0.897) | 20.15 | 0.077 | 0.862 | (0.808-0.916) | 18.22 | 0.082 |
| D5 | 0.788 | (0.709-0.867) | 16.17 | **0.015** | 0.794 | (0.724-0.864) | 13.64 | **0.003** |
| D7 | 0.703 | (0.611-0.796) | 18.37 | **<0.001** | 0.710 | (0.627-0.792) | 15.52 | **<0.001** |
| Tibialis anterior |  |  |  |  |  |  |  |  |
| D1 | 1.528 | (1.425-1.630) | 20.23 |  | 1.484 | (1.393-1.575) | 19.39 |  |
| D3 | 1.308 | (1.206-1.411) | 24.48 | **0.018** | 1.267 | (1.176-1.358) | 21.72 | **0.006** |
| D5 | 1.109 | (0.977-1.241) | 19.81 | **<0.001** | 1.106 | (0.989-1.224) | 18.67 | **<0.001** |
| D7 | 0.951 | (0.796-1.107) | 17.99 | **<0.001** | 0.932 | (0.794-1.071) | 11.97 | **<0.001** |

**Notes**: **CI:** confidence interval; **CV:** coefficient of variation; **D:** days of invasive mechanical ventilation; **p:** p-value.

**Online Resource S4**

**Table S4. Evolution of muscle thickness in upper limb muscles of mechanically ventilated patients with Covid-19 in the first week of admission to the intensive care unit.**

|  | **Upper right limb** | | | | **Upper left limb** | | | |
| --- | --- | --- | --- | --- | --- | --- | --- | --- |
| **Muscle thickness (mm)** | **Mean** | **95% CI** | **CV (%)** | ***p*** | **Mean** | **95% CI** | **CV (%)** | ***p*** |
| **Deltoid** |  |  |  |  |  |  |  |  |
| **D1** | 0.803 | (0.736-0.871) | 26.39 |  | 0.797 | (0.732-0.861) | 25.50 |  |
| **D3** | 0.712 | (0.644-0.779) | 27.83 | 0.232 | 0.711 | (0.646-0.776) | 25.40 | 0.252 |
| **D5** | 0.626 | (0.539-0.714) | 23.56 | **0.011** | 0.633 | (0.549-0.716) | 23.56 | **0.014** |
| **D7** | 0.588 | (0.486-0.691) | 22.15 | **0.004** | 0.595 | (0.497-0.694) | 23.96 | **0.005** |
| **Biceps brachialis (mm)** |  |  |  |  |  |  |  |  |
| **D1** | 1.288 | (1.171-1.405) | 26.83 |  | 1.259 | (1.164-1.355) | 23.40 |  |
| **D3** | 1.120 | (1.003-1.237) | 30.00 | 0.187 | 1.088 | (0.992-1.183) | 25.70 | 0.063 |
| **D5** | 0.968 | (0.818-1.119) | 31.79 | **0.007** | 0.946 | (0.823-1.070) | 24.09 | **0.001** |
| **D7** | 0.892 | (0.715-1.069) | 25.77 | **0.002** | 0.863 | (0.718-1.008) | 20.10 | **<0.001** |

**Notes**: **CI:** confidence interval; **CV:** coefficient of variation; **D:** days of invasive mechanical ventilation; **p:** p-value.

**Online Resource S5**

**Table S5. Evolution of muscle thickness in abdominal muscles of mechanically ventilated patients with Covid-19 in the first week of admission to the intensive care unit.**

| **Muscle thickness (mm)** | **Mean** | **95% CI** | **CV (%)** | ***p*** |
| --- | --- | --- | --- | --- |
| Rectus Abdominis |  |  |  |  |
| D1 | 0.701 | (0.638-0.764) | 27.54 |  |
| D3 | 0.632 | (0.569-0.695) | 27.12 | 0.420 |
| D5 | 0.560 | (0.479-0.641) | 27.35 | **0.037** |
| D7 | 0.494 | (0.399-0.590) | 30.57 | **0.003** |
| Transversus Abdominis |  |  |  |  |
| D1 | 0.290 | (0.258-0.323) | 29.95 |  |
| D3 | 0.256 | (0.224-0.288) | 32.36 | 0.445 |
| D5 | 0.228 | (0.186-0.270) | 40.97 | 0.093 |
| D7 | 0.205 | (0.156-0.254) | 48.33 | **0.024** |
| Internal Oblique |  |  |  |  |
| D1 | 0.453 | (0.413-0.494) | 24.96 |  |
| D3 | 0.391 | (0.350-0.432) | 27.84 | 0.144 |
| D5 | 0.336 | (0.2823-0.388) | 34.44 | **0.004** |
| D7 | 0.311 | (0.250-0.373) | 36.07 | **0.001** |
| External Oblique |  |  |  |  |
| D1 | 0.353 | (0.305-0.400) | 41.49 |  |
| D3 | 0.296 | (0.249-0.344) | 40.90 | 0.345 |
| D5 | 0.273 | (0.212-0.334) | 44.44 | 0.178 |
| D7 | 0.251 | (0.179-0.323) | 49.90 | 0.096 |

**Notes: CI:** confidence interval; **CV:** coefficient of variation; **D:** days of invasive mechanical ventilation; **p:** p-value.

**Online Resource S6**

**Table S6. Percentage of loss of muscle thickness in the lower, upper and abdominal limbs during 7 days of hospitalization in the ICU.**

| **Muscle thickness** | **D3-D1** | **D5-D1** | **D7-D1** |
| --- | --- | --- | --- |
| **Lower limbs** |  |  |  |
| Right quadriceps (%) | 16.28 | 30.12 | 41.18 |
| Left quadriceps (%) | 25.89 | 39.16 | 45.44 |
| Right rectus femoris (%) | 14.93 | 31.25 | 44.54 |
| Left rectus femoris (%) | 16.02 | 28.15 | 41.35 |
| Right vastus intermedius (%) | 15.63 | 28.29 | 37.94 |
| Left vastus intermedius (%) | 16.10 | 24.55 | 33.77 |
| Right medial gastrocnemius (%) | 11.50 | 20.48 | 29.20 |
| Left medial gastrocnemius (%) | 12.03 | 21.68 | 28.98 |
| Right lateral gastrocnemius (%) | 11.26 | 16.37 | 25.33 |
| Left lateral gastrocnemius (%) | 9.74 | 16.88 | 25.72 |
| Right anterior tibial (%) | 14.36 | 27.39 | 37.72 |
| Left tibialis anterior (%) | 14.63 | 25.48 | 37.19 |
| **Upper limbs** |  |  |  |
| Right deltoid (%) | 11.41 | 22.02 | 26.78 |
| Left deltoid (%) | 10.76 | 20.60 | 25.28 |
| Right biceps brachii (%) | 13.04 | 24.82 | 30.74 |
| Left biceps brachii (%) | 13.63 | 24.86 | 31.47 |
| **Abdominals** |  |  |  |
| Rectus abdominis (%) | 9.79 | 20.13 | 29.46 |
| Transversus abdominis (%) | 11.79 | 21.49 | 29.36 |
| Internal oblique (%) | 13.77 | 25.93 | 31.29 |
| External oblique (%) | 15.98 | 22.63 | 28.84 |

**Note: D:** days of invasive mechanical ventilation.

**Online Resource S7**

**Table S7. Percentage of loss of the cross-sectional area of the lower and upper limbs during 7 days of hospitalization in the ICU.**

| **Cross-sectional area** | **D3-D1** | **D5-D1** | **D7-D1** |
| --- | --- | --- | --- |
| **Lower limbs** |  |  |  |
| Right rectus femoris (%) | 14.22 | 27.79 | 44.93 |
| Left rectus femoris (%) | 15.68 | 25.49 | 42.28 |
| Right anterior tibial (%) | 12.14 | 25.65 | 35.54 |
| Left tibialis anterior (%) | 10.67 | 24.68 | 35.40 |
| **Upper limbs** |  |  |  |
| Right biceps brachii (%) | 11.09 | 22.97 | 29.28 |
| Left biceps brachii (%) | 11.77 | 25.09 | 30.68 |

**Note: D:** days of invasive mechanical ventilation.

**Online Resource S8**

**Table S8. Baseline characteristics compared between use of neuromuscular blockade.**

| **Variables** | **NMB (n=15)** | **No NMB (n=15)** | ***P**** |
| --- | --- | --- | --- |
| Male/Female (N, %) | 11/4 (73.3/26.7) | 10/5 (66.6/33.3) | 0.775 |
| Age, years (Mean ± SD) | 58 ± 17.6 | 61.7 ± 13.7 | 0.935 |
| APACHE II (Mean ± SD) | 24.3 ± 7.3 | 23.6 ± 4.9 | 0.713 |
| SAPS 3 (Mean ± SD) | 64.8 ± 8.7 | 63.5 ± 12.23 | 0.967 |
| VMI days (Mean ± SD) | 5.4 ± 0.5 | 4.6 ± 0.5 | 0.247 |
| Outcomes |  |  |  |
| Extubation < 7 days (N, %) | 3 (20) | 2 (13.3) | 0.766 |
| Tracheostomy < 7 days (N, %) | 1 (6.7) | 2 (13.3) | 0.766 |
| Mortality < 7 days (N, %) | 5 (33.3) | 8 (53.3) | 0.231 |
| Comorbidities |  |  |  |
| Systemic Arterial Hypertension (N. %) | 7 (46.7) | 7 (46.7) | 0.642 |
| Diabetes Mellitus (N, %) | 5 (33.3) | 8 (53.3) | 0.231 |
| Asthma (N, %) | 2 (13.3) | 0 | 0.241 |
| Obesity (N, %) | 6 (40) | 2 (13.3) | 0.107 |
| COPD (N, %) | 0 | 3 (20) | 0.112 |
| Heart diseases (N, %) | 1 (6.7) | 3 (20) | 0.299 |
| Kidney diseases (N, %) | 3 (20) | 0 | 0.112 |
| Other respiratory diseases (N, %) | 0 | 2 (13.3) | 0.241 |
| Human Immunodeficiency Virus (N, %) | 0 | 1 (6.7) | 0.500 |

**Notes:** NMB: neuromuscular blockade; APACHE II: Acute Physiology and Chronic Health Evaluation II; SAPS 3: Simplified Acute Physiology Score 3; COPD: Chronic Obstructive Pulmonary Disease; * T-test for independent samples for variables with normal distribution or Mann Whitney Test for variables with non-normal distribution. Chi-Square Test was used to compare proportions and corrected by Fisher's exact test (if n of cases < 5).

**Online Resource S9**

MANOVA test using NMB as a covariate in the model showed an effect of influence of NMB on muscle thickness loss and cross-sectional area reduction on specific muscle groups (Table S9).

**Table S9. The influence of NMB on US measurements on muscle thickness and cross-sectional area (MANOVA test using NMB as a covariate).**

| **Muscle thickness** | |
| --- | --- |
| Lower limbs | Right [F (1.86) = 10.63; p = 0.002] and left quadriceps [F(1.86) = 6.66; p = 0.012]; right [F (1.86) = 21.63; p < 0.001] and left vastus intermediate [F(1.86) = 22.34; p < 0.001]; right [F (1.86) = 13.07; p = 0.001] and left anterior tibialis [F(1.86) = 8.98; p = 0.004]; right [F (1.86) = 3.97; p = 0.049] and left lateral gastrocnemius [F(1.86) = 4.13; p = 0.045]. There was no difference for the rectus femoris and medial gastrocnemius muscles bilaterally. |
| Upper limbs | Right [F (1.86) = 19.82; p < 0.001] and left deltoid [F (1.86) = 18.37; p < 0.001]. There was no difference for the biceps brachii muscle bilaterally. |
| Abdominals | Rectus abdominis [F (1.86) = 5.48; p = 0.022]. internal oblique [F (1.86) = 13.20; p < 0.001] and external [F (1.86) = 16.46; p < 0.001]. and transversus abdominis [F (1.86) = 5.43; p = 0.022]. |
| **Cross-sectional area** | |
| Lower limbs | Right [F (1.86) = 19.60; p < 0.001] and left rectus femoris [F (1.86) = 22.32; p < 0.001]; right [F (1.86) = 12.73; p = 0.001] and left tibialis anterior [F (1.86) = 14.23; p < 0.001]. |
| Upper limbs | There was no difference for the left biceps brachii muscle bilaterally |

**ANCILLIARY ANALYSIS**

**Online Resource S10**

**Table S10.1. Proportion of muscle thickness loss higher than 15% by body segment over the 7 days after ICU admission.**

| **Muscle thickness loss**  **higher than 15%** | | D3-D1  (N=30) | D5-D1  (N=18) | D7-D1  (N=13) |
| --- | --- | --- | --- | --- |
| **Lower limbs** | | | |  |
| Right quadriceps | Yes (N, %) | 17 (57) | 16 (89) | 12 (92) |
|  | No (N, %) | 13 (43) | 2 (11) | 1 (8) |
|  | p | 0.585 | 0.001 | 0.003 |
| Left quadriceps | Yes (N, %) | 17 (57) | 17 (94) | 12 (92) |
|  | No (N, %) | 13 (43) | 1 (6) | 1 (8) |
|  | p | 0.585 | <0.001 | 0.003 |
| Right rectus femoris | Yes (N, %) | 16 (53) | 16 (89) | 12 (92) |
|  | No (N, %) | 14 (47) | 2 (11) | 1 (8) |
|  | p | 0.856 | 0.001 | 0.003 |
| Left rectus femoris | Yes (N, %) | 14 (47) | 16 (89) | 11 (85) |
|  | No (N, %) | 16 (53) | 2 (11) | 2 (15) |
|  | p | 0,856 | 0,001 | 0,022 |
| Right vastus intermedius | Yes (N, %) | 14 (47) | 16 (89) | 12 (92) |
|  | No (N, %) | 16 (53) | 2 (11) | 1 (8) |
|  | p | 0,856 | 0,001 | 0,003 |
| Left vastus intermedius | Yes (N, %) | 12 (40) | 15 (83) | 13 (100) |
|  | No (N, %) | 18 (60) | 3 (17) | 0 (0) |
|  | p | 0.362 | 0.008 | <0.001 |
| Right medial gastrocnemius | Yes (N, %) | 8 (27) | 9 (50) | 12 (92) |
|  | No (N, %) | 22 (73) | 9 (50) | 1 (8) |
|  | p | 0.016 | 1.000 | 0.003 |
| Left medial gastrocnemius | Yes (N, %) | 9 (30) | 11(61) | 11(85) |
|  | No (N, %) | 21(70) | 7 (39) | 2 (15) |
|  | p | 0.043 | 0.481 | 0.022 |
| Right lateral gastrocnemius | Yes (N, %) | 8 (27) | 10 (56) | 10 (77) |
|  | No (N, %) | 22 (73) | 8 (44) | 3 (23) |
|  | p | 0.016 | 0.815 | 0.092 |
| Left lateral gastrocnemius | Yes (N, %) | 8 (27) | 10 (56) | 11(85) |
|  | No (N, %) | 22 (73) | 8 (44) | 2 (15) |
|  | p | 0.016 | 0.815 | 0.022 |
| Right anterior tibial | Yes (N, %) | 16 (53) | 15 (83) | 12 (92) |
|  | No (N, %) | 14 (47) | 3 (17) | 1 (8) |
|  | p | 0.856 | 0.008 | 0.003 |
| Left tibialis anterior | Yes (N, %) | 14 (47) | 16 (89) | 13 (100) |
|  | No (N, %) | 16 (53) | 2 (11) | 0 (0) |
|  | p | 0.856 | 0.001 | <0.001 |
| **Upper limbs** |  |  |  |  |
| Right deltoid | Yes (N, %) | 8 (27) | 8 (44) | 12 (92) |
|  | No (N, %) | 22 (73) | 10 (56) | 1 (8) |
|  | p | 0.016 | 0.815 | 0.003 |
| Left deltoid | Yes (N, %) | 6 (20) | 8 (44) | 11(85) |
|  | No (N, %) | 24 (80) | 10 (56) | 2 (15) |
|  | p | 0.001 | 0.815 | 0.022 |
| Right biceps brachii | Yes (N, %) | 11 (37) | 15 (83) | 12 (92) |
|  | No (N, %) | 19 (63) | 3 (17) | 1 (8) |
|  | p | 0.200 | 0.008 | 0.003 |
| Left biceps brachii | Yes (N, %) | 12 (40) | 13 (72) | 11(85) |
|  | No (N, %) | 18 (60) | 5 (28) | 2 (15) |
|  | p | 0.362 | 0.096 | 0.022 |
| **Abdominals** |  |  |  |  |
| Rectus abdominis | Yes (N, %) | 6 (20) | 13 (72) | 12 (92) |
|  | No (N, %) | 24 (80) | 5 (28) | 1 (8) |
|  | p | 0.001 | 0.096 | 0.003 |
| Transversus abdominis | Yes (N, %) | 10 (33) | 14 (78) | 11(85) |
|  | No (N, %) | 20 (67) | 4 (22) | 2 (15) |
|  | p | 0.099 | 0.031 | 0.022 |
| Internal oblique | Yes (N, %) | 9 (30) | 11 (61) | 10 (77) |
|  | No (N, %) | 21 (70) | 7 (39) | 3 (23) |
|  | p | 0.043 | 0.481 | 0.092 |
| External oblique | Yes (N, %) | 10 (33) | 13 (72) | 10 (77) |
|  | No (N, %) | 20 (67) | 5 (28) | 3 (23) |
|  | p | 0.099 | 0.096 | 0.092 |

*Binomial test p-value was used to compare the proportions between the observed and expected observations set at 0.50 in each category.

**Table S10.2**. **Proportion of muscle cross-sectional area loss higher than 12% by body segment over the 7 days after ICU admission.**

| **Muscle cross-sectional area loss higher than 12%** | | D3-D1  (N=30) | | D5-D1  (N=18) | | D7-D1  (N=13) | |  |
| --- | --- | --- | --- | --- | --- | --- | --- | --- |
| **Lower limbs** | | | | | |  | |  |
| Right quadriceps | Yes (N, %) | | 18 (60) | | 16 (89) | | 13 (100) | |
|  | No (N, %) | | 12 (40) | | 2 (11) | | 0 (0) | |
|  | p | | 0.362 | | 0.001 | | <0.001 | |
| Left quadriceps | Yes (n. %) | | 20 (67) | | 17 (94) | | 13 (100) | |
|  | No (n. %) | | 10 (33) | | 1 (6) | | 0 (0) | |
|  | p | | 0.099 | | <0.001 | | <0.001 | |
| Right anterior tibial | Yes (n. %) | | 12 (40) | | 17 (94) | | 13 (100) | |
|  | No (n. %) | | 18 (60) | | 1 (6) | | 0 (0) | |
|  | p | | 0.362 | | <0.001 | | <0.001 | |
| Left tibialis anterior | Yes (n. %) | | 11 (37) | | 17 (94) | | 13 (100) | |
|  | No (n. %) | | 19 (63) | | 1 (6) | | 0 (0) | |
|  | p | | 0.200 | | <0.001 | | <0.001 | |
| **Upper limbs** |  | |  | |  | |  | |
| Right biceps brachii | Yes (n. %) | | 16 (53) | | 14 (78) | | 13 (100) | |
|  | No (n. %) | | 14 (47) | | 4 (22) | | 0 (0) | |
|  | p | | 0.856 | | 0.031 | | <0.001 | |
| Left biceps brachii | Yes (n. %) | | 15 (50) | | 14 (78) | | 11(85) | |
|  | No (n. %) | | 15 (50) | | 4 (22) | | 2 (15) | |
|  | p | | 1.000 | | 0.031 | | 0.022 | |

*Binomial test p-value was used to compare the proportions between the observed and expected observations set at 0.50 in each category.

**Online Resource S11**

The hypothesis of association between muscle mass loss higher than 15% and cross-sectional area decline higher than 12% with the outcomes ICU discharge/death were tested for all the 26 muscle groups, in which were only significant for muscle mass loss to right anterior tibialis and left biceps brachii between days 1 and 3 after ICU admission. (Online Resource Table S11.1)

**Table S11.1. Association between muscle mass loss higher than 15% and ICU discharge/death.**

|  | Outcome | | | Odds Ratio (CI 95%) | |  |
| --- | --- | --- | --- | --- | --- | --- |
|  | ICU discharge  N | Death  N | p | |  | |
| Right anterior tibial D3-D1 |  |  |  | |  | |
| No (N= 14) | 11 | 3 | 0.033* | | 6.11 (1.20-31.16) | |
| Yes (N=16) | 6 | 10 |  |  |  |  |
| All (N=30) | 17 | 13 |  | |  | |
| Left biceps brachii D3-D1 |  |  |  | |  | |
| No (N=18) | 14 | 4 | 0.008* | | 10.5 (1.89-58.36) | |
| Yes (N= 2) | 3 | 9 |  |  |  | |
| All (N=30) | 17 | 13 |  | |  | |

***** Chi-Square Test was used to compare proportions and corrected by Fisher's exact test (if n of cases < 5).

Several predictors, defined by muscle mass loss higher than 15% and cross-sectional area decline higher than 12% for all muscle groups, were tested by the automatic linear modelling for ICU discharge/death as target variable. Only 15%-muscle-mass decline of left biceps brachii as predictor variable was significantly influent (p= 0.002) (Table S11.2).

**Table S11.2.** **Regression model for ICU discharge/death as target variable with muscle mass thickness loss higher than 15%.**

Final regression model

| Model | R | R² | R² ajusted | S.E. | Change R² | Change F | Sig. Change F |
| --- | --- | --- | --- | --- | --- | --- | --- |
| 1 | 0.523^a^ | 0.274 | 0.220 | 0.445 | 0.274 | 5.094 | 0.013 |
| 2 | 0.522^b^ | 0.272 | 0.246 | 0.438 | -0.0002 | 0.064 | 0.803 |

a. Predictors: (Constant), Transversus Abdominal %15th D3-D1

loss, Left Biceps Brachii %15th D3-D1loss

b. Predictors: (Constant), Left Biceps Brachii %15th D3-D1loss

Notes: S.E. = Standard error; Sig. = significance.

| **ANOVA^a^** | | | | | | |
| --- | --- | --- | --- | --- | --- | --- |
| Model | | Sum of Squares | df | Mean Square | Z | p |
| 1 | Regression | 2.018 | 2 | 1.009 | 5.094 | 0.013^b^ |
|  | Residual | 5.348 | 27 | 0.198 |  |  |
|  | Total | 7.367 | 29 |  |  |  |
| 2 | Regression | 2.006 | 1 | 2.006 | 10.475 | 0.002^c^ |
|  | Residual | 5.361 | 28 | 0.191 |  |  |
|  | Total | 7.367 | 29 |  |  |  |
| a. Dependent Variable: Death/ICU discharge | | | | | | |
| b. Predictors: (Constant), Transversus Abdominal %15th D3-D1  loss, Left Biceps Brachii %15th D3-D1loss | | | | | | |
| c. Preditors: (Constant), Left Biceps Brachii %15th D3-D1loss  Notes: df = degrees of freedom; p = p-value. | | | | | | |

| Model term | Coefficient | S.E. | p | 95% Confidence Interval | | Importance |
| --- | --- | --- | --- | --- | --- | --- |
|  |  |  |  | Lower | Upper |  |
| Intercept | 1.854 | 0.131 | <0.001 | 1.586 | 2.123 |  |
| Left Biceps Brachii %15th D3-D1loss (yes) | -0.458 | 0.159 | 0.008 | -0.784 | -0.133 | 0.675 |
| Left Biceps Brachii %15th D3-D1loss (no) | 0ª |  |  |  |  | 0.675 |
| Transversus Abdominal %15th D3-D1loss  (yes) | -0.312 | 0.156 | 0.055 | -0.632 | 0.007 | 0.325 |
| Transversus Abdominal %15th D3-D1loss  (no) | 0ª |  |  |  |  | 0.325 |

ª This parameter is set to zero because it is redundant.

Notes: S.E. = Standard error; p = p-value.

**3)** By testing a regression model for death/ICU discharge as target variable and replacing the predictor variables for the percent changes of all muscle groups between every possible days intervals (D3-D1, D5-D1 and D7-D1). We reached a greater goodness of fit by adjusted R squared of 0.861. The adjusted model ranked the left biceps brachii thickness change with top importance (0.309) and by adding the following variables listed down on the table, the model reduced its explanatory power.

| Source | Sum of squares | df | Mean Square | p |
| --- | --- | --- | --- | --- |
| Corrected Model | 4.984 | 8 | 0.623 | <0.001 |
| Left Biceps Brachii %th D3-D1 | 2.513 | 1 | 2.513 | <0.001 |
| Right Biceps Brachii %th D3-D1 | 1.536 | 1 | 1.536 | <0.001 |
| Right Rectus Femoris %th D3-D1 | 0.993 | 1 | 0.993 | <0.001 |
| Right anterior tibialis % CSA D3-D1 | 0.795 | 1 | 0.795 | <0.001 |
| Left rectus femoris %th D7-D1 | 0.764 | 1 | 0.764 | <0.001 |
| Transversus Abdominis %th D3-D1 | 0.503 | 1 | 0.503 | 0.003 |
| Rectus Abdominis %th D5-D1 | 0.233 | 1 | 0.233 | 0.025 |
| Residual | 0.470 | 13 | 0.036 |  |
| Corrected Total | 5.455 | 21 |  |  |

Notes: df = degrees of freedom; p = p-value.

| Model term |  |  |  | 95% Confidence Interval | |  | |
| --- | --- | --- | --- | --- | --- | --- | --- |
|  | Coefficient | S.E. | p | Lower | Upper | Importance |  |
| Intercept | 3.772 | 0.394 | <0.001 | 2.922 | 4.623 |  |  |
| Left Biceps Brachii %th D3-D1 | -0.113 | 0.014 | <0.001 | -0.143 | -0.084 | 0.309 |  |
| Right Biceps Brachii %th D3-D1 | 0.067 | 0.010 | <0.001 | 0.045 | 0.089 | 0.189 |  |
| Right Rectus Femoris %th D3-D1 | 0.026 | 0.005 | <0.001 | 0.015 | 0.037 | 0.122 |  |
| Right anterior tibialis CSA D3-D1 | 0.046 | 0.010 | <0.001 | 0.025 | 0.068 | 0.098 |  |
| Left rectus femoris %th D7-D1 | 0.022 | 0.005 | 0.001 | 0.012 | 0.033 | 0.094 |  |
| Transversus Abdominis %th D3-D1 | -0.026 | 0.007 | 0.003 | -0.041 | -0.011 | 0.062 |  |
| Rectus Abdominis %th D5-D1 | 0.015 | 0.006 | <0.001 | 0.002 | 0.027 | 0.029 |  |

Notes: S.E. = Standard error; p = p-value.

**Online Resource S12**

**Regression modeling for the “death/ICU discharge” target variable.**

All the baseline clinical characteristics and muscle groups thickness and cross-sectional area changes between D3-D1, D5-D1 and D7-D1 were tested as predictors variables either separately and by blocks (Model Selection Method: Forward Stepwise; Information Criterion: -54.820. The information criterion is used to compare to models. Models with smaller information criterion values fit better).

The best automatic linear model was selected with an adjusted R² of 0.888 with the predictors levels ranked based on their importance level, having the biceps brachii muscle thickness changes between day 3 and day 1 in the first place, followed by left rectus femoris muscle thickness change between day 7 and day 1 and SAPS 3 score (Table S12).

**Table S12. Regression modeling for the “death/ICU discharge” target variable.**

| Source | Sum of squares | df | Mean Square | p |
| --- | --- | --- | --- | --- |
| Corrected Model | 5.048 | 8 | 0.631 | <0.001 |
| Left Biceps Brachii %th D3-D1 | 2.439 | 1 | 2.439 | <0.001 |
| Right Biceps Brachii %th D3-D1 | 1.304 | 1 | 1.304 | <0.001 |
| Left rectus femoris %th D7-D1 | 0.359 | 1 | 0.359 | 0.005 |
| SAPS3 | 0.299 | 1 | 0.299 | 0.009 |
| Right Biceps Brachii %th D7-D1 | 0.167 | 1 | 0.167 | 0.038 |
| Residual | 0.406 | 13 | 0.031 |  |
| Corrected Total | 5.455 | 21 |  |  |

| Model term | Coefficient | p | Importance |
| --- | --- | --- | --- |
| Intercept | 1.512 | 0.001 |  |
| Left Biceps Brachii %th D3-D1 | -0.095 | <0.001 | 0.419 |
| Right Biceps Brachii %th D3-D1 | 0.061 | <0.001 | 0.224 |
| Left rectus femoris %th D7-D1 | 0.017 | 0.005 | 0.062 |
| SAPS3 | 0.013 | 0.009 | 0.051 |
| Right Biceps Brachii %th D7-D1 | 0.018 | 0.038 | 0.029 |

Notes: df = degrees of freedom; p = p-value.
